# Antitumor Activity of Radiation Therapy Combined with Checkpoint Kinase Inhibition in SHH/*p53*-Mutated Human Medulloblastoma

**DOI:** 10.3390/ijms26062577

**Published:** 2025-03-13

**Authors:** Zuzana Kuchařová, Annegret Glasow, Rolf-Dieter Kortmann, Ina Patties

**Affiliations:** 1 Department of Radiation Oncology, Leipzig University, Stephanstraße 9A, 04103 Leipzig, Germany; zuzana.kucharova@medizin.uni-leipzig.de (Z.K.); annegret.glasow@medizin.uni-leipzig.de (A.G.); rolf-dieter.kortmann@uk-essen.de (R.-D.K.); 2 Comprehensive Cancer Center Central Germany (CCCG), Liebigstraße 22, 04103 Leipzig, Germany

**Keywords:** cell cycle checkpoint inhibition, checkpoint kinase 1, medulloblastoma, radiation therapy, orthotopic mouse model, prexasertib, SAR-020106, DNA damage

## Abstract

Medulloblastoma (MB) is one of the most common malignant pediatric brain tumors. Current therapy results in a poor prognosis for high-risk SHH/*p53*-mutated MB, emphasizing the importance of more effective therapeutic strategies. Here, we investigated the potential radiosensitizing effects of the checkpoint kinase inhibitors (Chk-is) prexasertib (Chk1/2) and SAR-020106 (Chk1) in human SHH/*p53*-mutated MB in vitro and in vivo. UW228 and DAOY cells were treated with Chk-is and irradiation (RT). Metabolic activity, proliferation, and apoptosis were determined at d3, and long-term clonogenicity was determined at d14. DNA damage was assessed after 1, 24, and 72 h. Patient-derived SHH/*p53*-mutated, luciferase-transfected MB cells were implanted orthotopically into NSG mice (d0). Fractionated therapy (daily, d7–11) was applied. Body weight (BW) was documented daily, tumor growth weekly, and proliferation at d42. In vitro, Chk-is exhibited a dose-dependent reduction in metabolic activity, proliferation, and clonogenicity and increased apoptosis. A combination of Chk-is with RT enhanced these antitumor effects, including proliferation, apoptosis, and clonogenicity, and increased residual DNA damage compared to RT alone. In vivo, tumor growth was delayed by Chk-is alone. Low-dose prexasertib enhanced RT-induced tumor growth inhibition. High-dose prexasertib and SAR-020106 showed opposite effects, at least at later time points (*n* = 3). BW assessments revealed that the treatment was well tolerated. Our data indicate a potential benefit of Chk-is in combination with RT in SHH/*p53*-mutated MB. However, high-dose Chk-is may compromise the RT effect, possibly through anti-proliferative activity. Furthermore, we demonstrate, for the first time, the intracranial antitumor activity of the Chk1-specific inhibitor SAR-020106.

## 1. Introduction

Medulloblastoma (MB) is one of the most common malignant brain tumors in children with a median age between five and nine years [1]. According to the 2021 WHO classification of CNS tumors, four molecular MB subgroups are defined, differing in prognosis and thereon-adapted therapy regimes [2].

Sonic hedgehog-activated and *TP53*-mutant (SHH/*p53*-mut) MB is a high-risk sub-group showing enhanced therapy resistance and has one of the worst prognoses among the MB subgroups [3,4].

The current treatment for SHH/*p53*-mut MB patients consists of maximum safe surgical resection, *TP53* mutation-dependent (somatic or germline) radiation therapy (RT), and chemotherapy according to the SIOP-PNET5-MB-SHH-TP53 trial protocol [5]. High-dose-intensity regimes and RT, as well as chemotherapy, cause devastating (long-term) adverse effects, which might decrease the quality of life. The disruption of white matter development and reduced hippocampal neurogenesis after high-intensity therapy are known biological causes of neurological and sensory impairments and endocrine deficits in adolescent patients [6,7].

Therefore, there is an urgent clinical need to identify new approaches for the treatment of high-risk MB, improving patient outcomes while minimizing adverse therapy effects. An appropriate strategy is to increase (tumor cell-specific) RT efficacy by inhibiting the repair of RT-induced DNA damage. As a consequence, lower RT doses and/or prolonged overall survival (OS) could be achieved.

The unrestricted G1/S transition in *TP53*-mutated cells circumvents DNA repair in G1 and enforces it at later cell cycle stages [8]. Therefore, the SHH/*p53*-mut MB subgroup is particularly vulnerable to DNA damage repair blockade caused by G2/M checkpoint abrogation, leading to DNA damage accumulation and enhanced cell death. The checkpoint kinases Chk1 and Chk2 are responsible for DNA damage-induced cell cycle arrest/prolongation, allowing for DNA repair [9]. Notably, Chk1 plays a crucial role in activating the G2/M checkpoint after phosphorylation by ATR (ataxia telangiectasia and Rad3-related protein), a DNA damage sensor protein. When inhibited, the cell enters mitosis with fragmented chromosomes, resulting in cell death. Additionally, Chk1 inhibition leads to an unscheduled increase in DNA replication forks, generating more DNA damage and resulting in replication catastrophe [10,11]. Moreover, the constitutive higher expression of phosphorylated Chk1 compared to normal brain tissue has been found in medulloblastoma patient samples [12]. Several Chk-is have been examined in clinical trials but have not been approved for clinical application because of off-target activities [13,14]. Here, we compare the in vitro and in vivo characteristics of two Chk-is with an emphasis on Chk1 inhibition.

Prexasertib (PRX) is an ATP-competitive inhibitor of Chk1 and, to a lesser extent, of Chk2 [10]. PRX monotherapy induces DNA damage and tumor cell death in vitro and tumor growth delay in vivo [10,15]. A combination of PRX with RT and cisplatin in head and neck squamous cell carcinoma (HNSCC) has shown enhanced antitumor effects [11,16]. In MB, PRX sensitizes tumor cells to genotoxic drugs like cyclophosphamide, cisplatin, or gemcitabine in vitro and in vivo. The strong chemosensitizing activity of PRX has been shown to reduce tumor burden and increase survival in high-risk MB-bearing mice [17]. However, the effect of PRX combined with RT in MB is still unknown. In clinical trials, PRX is already under investigation, administered as a monotherapy or in combination with cytotoxic agents. Also, in advanced MB patients, the SHH subgroup included, the efficacy of PRX in combination with gemcitabine or cyclophosphamide is currently being evaluated [18].

In contrast to PRX, SAR-020106 (SAR) is highly selective for Chk1 inhibition [19]. In colon carcinoma xenografts, it is able to enhance the efficacy of genotoxic drugs like irinotecan and gemcitabine [19,20]. Furthermore, in HNSCC and colorectal cancer, SAR sensitizes tumor cells to RT-induced damage in vitro and in vivo [19,20,21]. In glioblastoma cells, we have already shown the chemo- and radiosensitizing effect of SAR, prolonging genotoxic-induced DNA damage repair and reducing clonogenic long-term survival [22].

Despite improvements in the treatment of MB, the prognosis of pediatric patients with the SHH/*p53*-mut subtype remains poor. This might partly be due to the impairment of the p53-Chk2 pathway, resulting in limited DNA damage repair and apoptosis at the G1/S cell cycle checkpoint. This led us to the hypothesis that the additional inhibition of Chk1, another essential DNA damage repair protein, with Chk-is might further enhance DNA damage accumulation, specifically in these *p53*-mutated MB cells, finally resulting in tumor cell death, e.g., through p53-independent apoptosis, replication catastrophe, and/or mitotic catastrophe [10,11].

Therefore, the current study examines, for the first time, the combinatory effects of RT with the Chk-is PRX and SAR in human SHH/*p53*-mut MB. In vitro, the influence on DNA damage repair, long-term clonogenic tumor cell survival, apoptosis, proliferation, and metabolic activity was analyzed in two SHH/*p53*-mut MB cell lines. In vivo, an orthotopic patient-derived SHH/*p53*-mut MB xenograft mouse model was used to assess tumor growth via bioluminescence imaging (BLI) and proliferation index through Ki-67 staining of tumor tissue. Therefore, DNA damage was induced by ionizing radiation, as it is an inherent part of MB standard therapy.

These investigations will outline a possible clinical opportunity for the therapy of high-risk SHH/*p53*-mut MB patients with adjuvant Chk1 inhibition.

## 2. Results and Discussion

**Figure 1:**
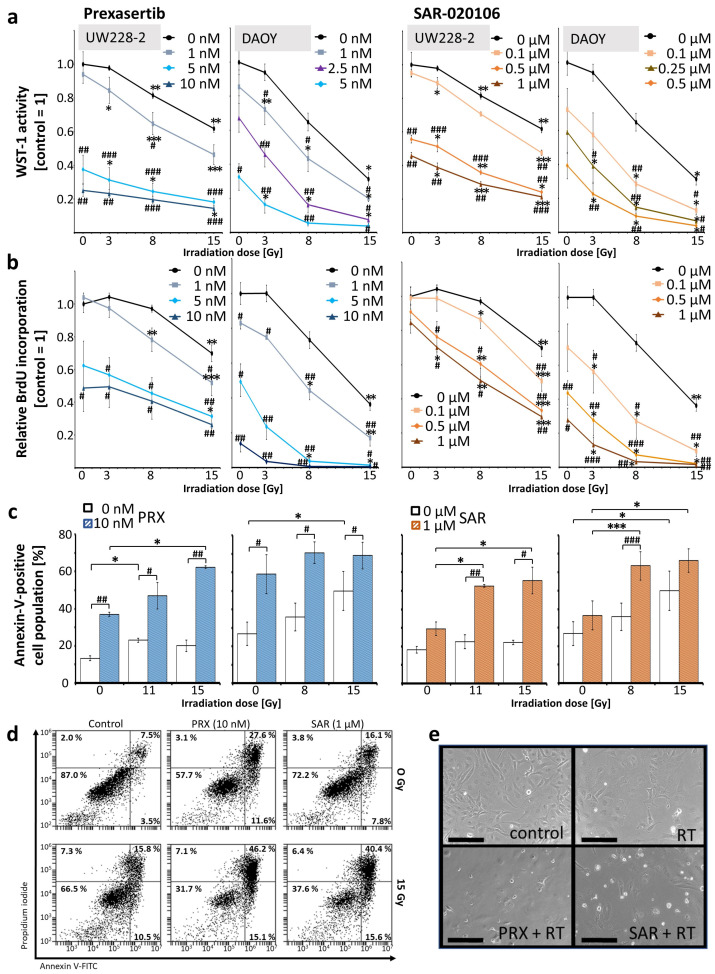
Effects of combined Chk inhibition and RT on metabolic activity, proliferation, and apoptosis of UW228 and DAOY cells 72 h after treatment. (**a**) Metabolic activity measured by WST-1 assay; (**b**) proliferation measured by BrdU ELISA. Data in line charts (**a**,**b**) represent mean ± standard error of mean (SEM) of three independent experiments performed in triplicate. (**c**,**d**) Apoptosis measured by Annexin-V flow cytometry. (**c**) Annexin-V-positive cell fraction is presented as mean ± SEM measured in three independent experiments performed as single replicates. Statistical significance compared to RT-only (also sham-RT) is indicated by number signs (#, *p* ≤ 0.05; ##, *p* ≤ 0.01; ###, *p* ≤ 0.001) and compared to drug-only (also placebo) by asterisks (*, *p* ≤ 0.05; **, *p* ≤ 0.01; ***, *p* ≤ 0.001). (**d**) Representative dot blots from Annexin-V assay of UW228 cells. (**e**) Phase contrast images of differentially treated (15 Gy RT; 10 nM PRX; 1 µM SAR) UW228 cells: 20-fold magnification; scale bar = 200 µm.

First, we determined the antitumor activity and mechanisms of two Chk-is in the human SHH/*p53*-mut MB cell lines UW228 and DAOY in vitro. The short-term in vitro effects of Chk inhibition by PRX (Chk1/2) or SAR (Chk1) combined with RT on metabolic activity, proliferation, and apoptotic cell death 72 h after a single-time treatment are shown in Figure 1.

The application of PRX or SAR alone led to a concentration-dependent decrease in metabolic activity and proliferation in UW228 and DAOY cells, with slightly higher sensitivity in DAOY cells (Figure 1a,b). Significant effects on proliferation were seen at 1 nM PRX (DAOY, *p* ≤ 0.05) and 10 nM PRX (UW228, *p* ≤ 0.05). For SAR, at least 500-fold higher concentrations were needed to reach similar effects (Figure 1b). PRX (10 nM) also enhanced the apoptotic cell fraction (percentage of Annexin-V-positive cells) in UW228/DAOY cells by 24/32% versus the untreated control (16/27%; *p* ≤ 0.05). SAR was less toxic and had only minor effects (an 11/10% increase versus the control, n.s.) (Figure 1c).

In accordance with the known therapy resistance of SHH MB [23], high RT doses were necessary to significantly reduce metabolic activity (8–15 Gy) and proliferation (15 Gy) in both cell lines. Only in DAOY cells was minor RT-induced short-term cell death (apoptosis) induction found (15 Gy, *p* ≤ 0.05).

As hypothesized, pretreatment with Chk-is enhanced the RT effects. Metabolic activity and proliferation were significantly reduced at 3 Gy when combined with Chk-is, and apoptotic fraction was enhanced at 11 Gy (UW228) and 8 Gy (DAOY). The strongest effects were detected after combinatorial treatment with 15 Gy. Therefore, the RT-induced apoptotic fraction was enhanced by 42% in UW228 and 19% in DAOY after PRX and by 34% in UW228 and 16% in DAOY after SAR, showing, again, DAOY cells to be slightly more sensitive than UW228 cells (Figure 1c,d). Phase contrast photographs revealed morphological changes like cell rounding/detachment and disaggregation were strongest in the combinatory treatment group, going along with enhanced cell death (Figure 1e).

Our data reveal, for the first time, the pronounced radiation-induced antitumor effects of Chk-is in SHH/*p53*-mut MB cells, and are in line with recent in vitro and preclinical findings demonstrating the single-agent, as well as sensitizing efficacy, of PRX and SAR when combined with genotoxic treatment in different tumor entities [10,11,15,16,19,21,24,25,26,27,28,29], including DNA-damaging chemotherapy in MB [17]. Therefore, DNA damage induction with simultaneous Chk1 inhibition often enhanced cell death, especially in p53-deficient cells where the ATM–Chk2–p53 signaling pathway is already disabled, impeding DNA repair through non-homologous end-joining [10,21,30]. In p53-deficient cells, p53-independent apoptosis can be induced via Caspase-2, which is additionally enhanced by Chk1 inhibition [31]. Accordingly, we previously demonstrated that the Chk1-i SAR enhances the cytotoxic effects of genotoxic drugs and/or radiation more strongly in p53-deficient cells than in p53-wildtype glioblastoma cells [22].

**Figure 2:**
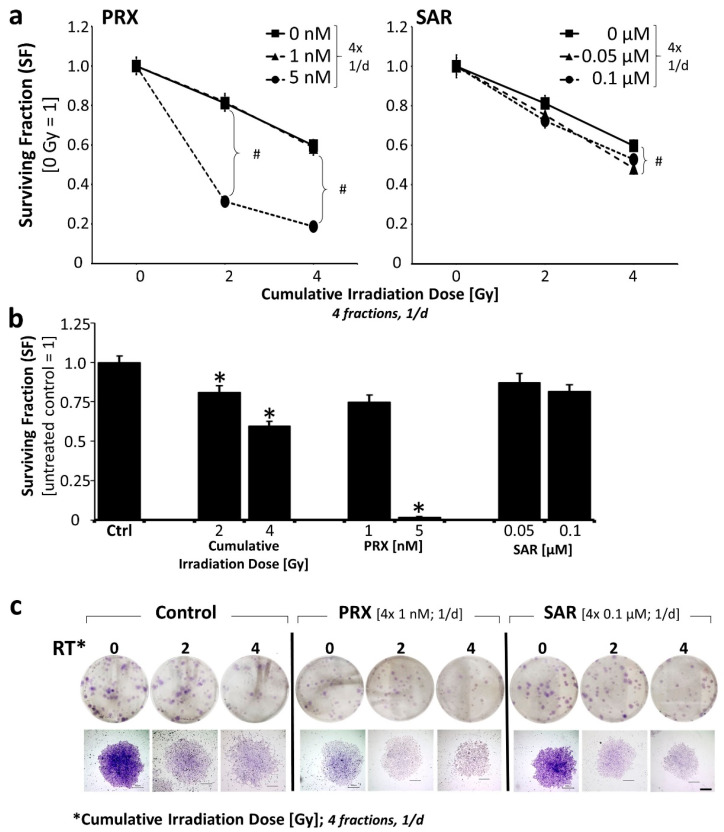
Effects of combined Chk inhibition and RT on clonogenic survival of UW228 cells 14 d after fractionated treatment (4 fractions, 1/d). (**a**) Clonogenic survival after combined treatment relative to sham-irradiated control (=1). Significant effects versus RT alone (#, *p* ≤ 0.05) were found at 4 × 0.5 Gy and 4 × 1 Gy for 5 nM PRX (SF = 0.006 ± 0.001 and 135-fold; SF = 0.003 ± 0.001 and 200-fold) and at 4 × 0.5 Gy for 0.1 µM SAR (SF = 0.59 ± 0.04; 1.4-fold). (**b**) Clonogenic survival after single treatments: surviving fraction of UW228 cells decreased after fractionated RT (SF = 0.81 ± 0.04 at 4 × 0.5 Gy and 0.60 ± 0.03 at 4 × 1 Gy). PRX reduced SF to 0.75 ± 0.04 (1 nM) and 0.018 ± 0.004 (5 nM). SAR diminished SF to 0.87 ± 0.06 (0.05 µM) and 0.82 ± 0.04 (0.1 µM). Significance compared to untreated/sham-irradiated control (=1) is indicated by asterisks (*, *p* ≤ 0.05). (**a**,**b**) Data represent mean ± SEM of three independent experiments performed in sextuplicate. (**c**) Representative photographs of grown colonies on 6-well plates (**top**) and single colonies (**bottom**); 100 cells were initially seeded. Scale bar = 500 µM.

Clonogenic long-term survival was investigated exemplarily in the less responsive UW228 MB cell line 14 d after four repetitive treatment days (Figure 2). Therefore, about 25% and 18% reductions in SF were achieved by PRX (4 × 1 nM) and SAR (4 × 0.1 µM) alone, showing, again, the higher activity of PRX versus SAR. A further increase in PRX concentration (4 × 5 nM) reduced the SF massively by 92% (Figure 2b).

The combination of Chk-is with RT revealed the radiosensitizing effect of PRX (4 × 5 nM) and SAR (4 × 0.1 µM) on clonogenic tumor cell death, resulting in fewer less dense colonies (Figure 2c).

**Figure 3:**
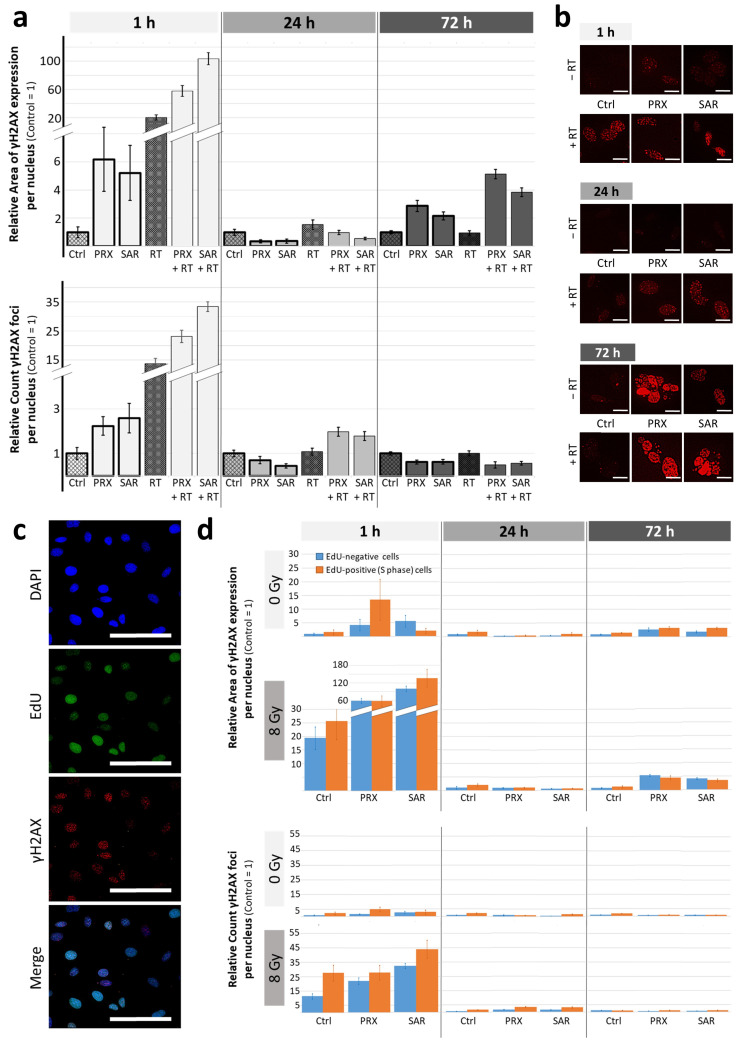
DNA damage in UW228 cells after combined Chk inhibition (5 nM PRX or 0.5 µM SAR) and RT (8 Gy). (**a**) Relative area of γH2AX expression/nucleus and relative number of γH2AX foci/nucleus 1, 24, and 72 h after treatment. Data represent mean ± SEM of at least 50 analyzed cell nuclei from one experiment. (**b**) Representative photographs of DNA double-stand breaks indicated by γH2AX immunofluorescence staining 1, 24, and 72 h after treatment. Scale bars = 10 µm. (**c**) Representative images of immunofluorescence-stained nuclei (blue), EdU (green), and γH2AX protein (red); scale bars = 100 µm. (**d**) Relative area of γH2AX expression/nucleus and relative number of γH2AX foci/nucleus in EdU-positive S/G2 phase and EdU-negative cells 1, 24, and 72 h after treatment. Data represent mean ± SEM of at least 50 analyzed cell nuclei from one experiment.

To obtain more insight into the effects of Chk1/2 (PRX) and selective Chk1 (SAR) inhibition regarding the repair of RT-induced DSB, we examined DNA damage proteins using γH2AX immunofluorescence and γH2AX/pRPA Western blot (WB) 1, 24, and 72 h after treatment. Exemplarily analysis of DNA damage based on γH2AX area and γH2AX foci per nucleus confirmed the accumulation of unrepaired DNA damage during ongoing cell cycles as a major acting mechanism of both Chk-is in MB (Figure 3a,b).

The induction of DNA damage was detected 1 h after the Chk-i and/or RT treatments. Therefore, the highest induction levels were seen after combinatory treatment with an enhanced foci number, as well as enlarged areas of γH2AX expression/nucleus, the latter possibly representing condensed DNA damage. After 24 h, γH2AX foci per nucleus were enhanced most after the combined treatment. This goes along with our previous studies of glioblastoma cells showing about 2-fold elevated residual RT-induced DNA damage 24 h after combined treatment with SAR and RT [22]. Also, others have shown the DNA damage-enhancing effect of SAR combined with chemo- or radiotherapy on colon; head and neck; and lung cancer cell lines [19,21]. Likewise, PRX monotherapy has been found to induce DNA breakage in cervix and head and neck cancer cells [10,25].

Even after 72 h, the Chk-i treatment enlarged the relative area of γH2AX expression/nucleus (PRX, 2.9-fold; SAR, 2.2-fold) compared to the control (=1; total area/nucleus 14 ± 2%). The combinatorial treatment of Chk-i with RT further enhanced the relative area of γH2AX expression/nucleus (PRX, 5.4-fold; SAR, 4.1-fold) compared to RT alone (total area/nucleus, 13 ± 2%) at 72 h (Figure 3a,b). This indicates persistent DNA damage accumulation caused by Chk-i over at least 1–2 cell cycles (doubling time approx. 35 h).

For a specific evaluation of Chk-i effects on DNA damage induction during the cell cycle, γH2AX staining was conducted on EdU-positive (S and G2/M phase) and EdU--negative cells (Figure 3c,d). Thus, we found the PRX single treatment induced DNA damage more strongly in EdU-positive cells at 1 h, affirming findings by King et al. showing PRX-induced DNA damage during the S phase in cervix cancer cells [10].

**Figure 4:**
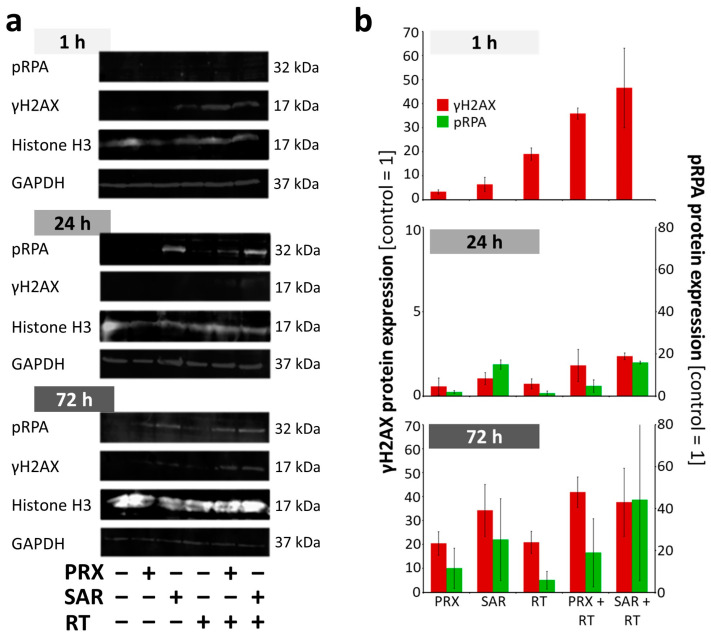
Western blot analyses of pRPA and γH2AX in UW228 cells after combined Chk inhibition (5 nM PRX or 0.5 µM SAR) and RT (8 Gy). (**a**) Representative Western blots of pRPA, γH2AX, and loading controls (Histone H3; GAPDH) stained with fluorescent-labeled secondary antibodies. (**b**) Quantitative Western blot analyses of pRPA and γH2AX fluorescence intensities. Data represent mean ± SEM of two blotting membranes from one experiment. Data are corrected to appropriate loading controls (Histone H3 and GAPDH) and normalized to the untreated control.

In a second independent experiment, WB confirmed the induction of γH2AX by RT and a slight induction by Chk-i at 1 h (Figure 4a,b). The combinatory treatment resulted in an enhanced initial γH2AX (1 h) and a slightly enhanced residual γH2AX at 24 h. At 72 h, γH2AX expression rose again, confirming the microscopic analysis in Figure 3a,b. PRPA, a single-strand break protein, was not detectable at 1 h and showed only a slight induction by RT and Chk-i at 24 h; combinatory treatments enhanced these effects. At 72 h, similar to γH2AX expression, pRPA levels increased in all treatment groups compared to 24 h. A reduction in single- and double-strand breaks at 24 h indicates DNA damage repair; however, this could be inaccurate due to the abrogation of Chk-dependent cell cycle arrest. Cells might then enter the next cell cycle, accumulating DNA mistakes and leading, again, to DNA damage during DNA synthesis enhanced by Chk-i, corresponding to the observed high levels of DNA damage protein expression at 72 h. Taken together, we could demonstrate that the repair of RT-induced DNA damage can be efficiently inhibited by PRX and SAR and is not restricted to S or G2/M cell cycle phases.

**Figure 5:**
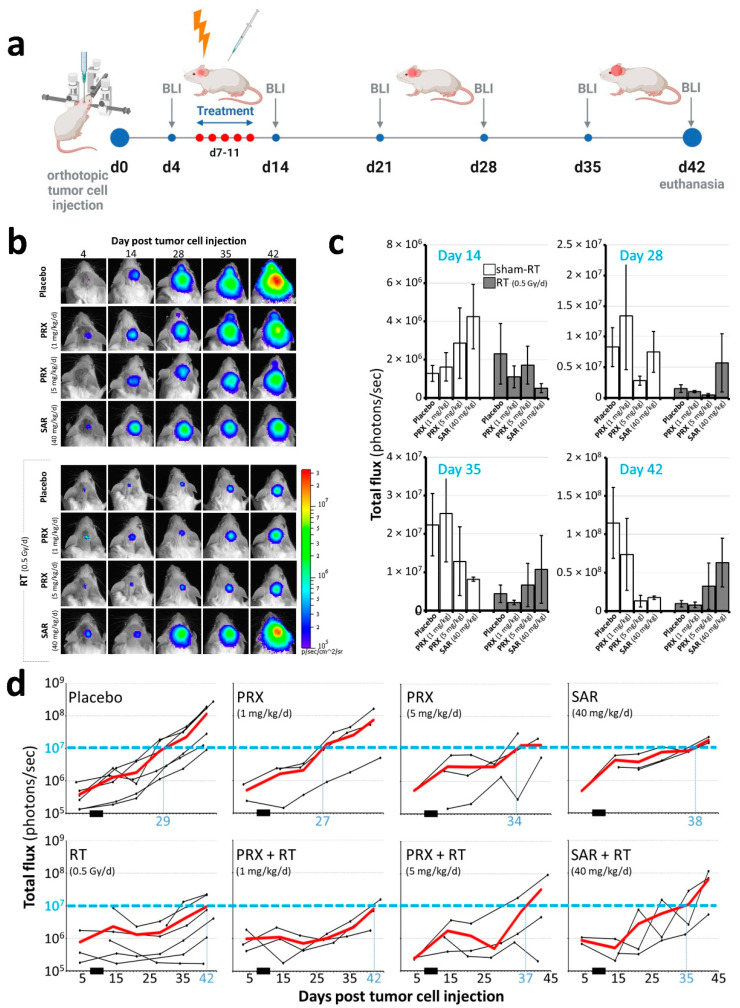
Tumor growth of SHH/*p53*-mut MB after fractionated therapy (5×, daily) with PRX (1 or 5 mg/kg/d) or SAR (40 mg/kg/d) and RT (0.5 Gy/d). (**a**) Workflow of in vivo procedures. Treatment comprised PRX (1 or 5 mg/kg/d, s.c.) or SAR (40 mg/kg/d, i.p.) and RT (0.5 Gy/d) 2 h later. Created with BioRender.com (accessed on 1 August 2024). (**b**) Representative bioluminescence images. (**c**) Total flux at d14, 28, 35, and 42 after tumor inoculation. (**d**) Total flux of single mouse BLI measurements and mean of all (red line). Treatment window is indicated by a black bar on x-axis. Blue dotted line visualizes the absolute total flux (10^7^) of the RT group at d42.

To validate the promising in vitro data in vivo, we performed a proof-of-principle study with at least three mice per treatment group using an orthotopic SHH/*p53*-mut MB PDX model (Figure 5a). A limitation of this observational study is the low number of animals (*n* = 3 per time point and treatment), which restricts the statistical power. Estimated in vivo doses are in range with (PRX) or above (SAR) the efficient in vitro concentrations (see Methods section). For SAR, the brain barrier penetration capability is still unknown. To the best of our knowledge, this is the first assessment of its potential intracranial antitumor activity.

**Figure 6:**
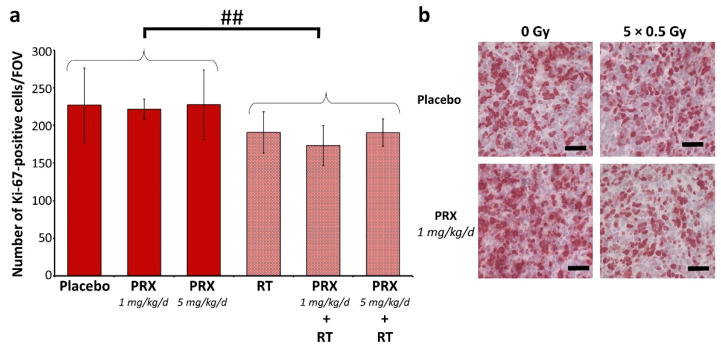
Proliferation index of SHH/*p53*-mut MB four weeks after fractionated therapy (5×, daily) with PRX and RT (0.5 Gy/d). (**a**) Mean number of Ki-67-positive cells per field of view (FOV) in the most proliferative tumor area. Data represent mean ± SEM from three mice with three analyzed FOVs. Significance analyses of all RT-treated groups (*n* = 9) compared with all sham-RT-treated groups (*n* = 9) are indicated by number signs (##, *p* ≤ 0.01). (**b**) Representative photographs of Ki-67-stained cells: 400-fold magnification; scale bars = 50 µm.

The tumor mass of placebo-treated animals grew continually over time and was enhanced 301-fold at d42 (euthanasia) versus d4 (Figure 5b–d). SAR monotherapy initially enhanced tumor growth at d14 (3.32-fold), presumably due to cell cycle acceleration caused by G2/M checkpoint abrogation. This was followed by growth inhibition compared to the control (d28–42; *n* = 3), assumedly due to DNA damage accumulation, as shown in vitro (Figure 3). PRX alone induced similar effects, with stronger effects at 5 mg/kg versus 1 mg/kg (*n* = 3). RT alone did not cure the animals, as BLI evaluation revealed the progression of tumor growth on d35 (Figure 5b,d). Only low-dose PRX (1 mg/kg) showed a tumor mass reduction in addition to RT at all executed time points. In contrast, high-dose PRX (5 mg/kg) and SAR seemed to exhibit the opposite effect, causing increased tumor mass if combined with RT. This might be explained (1) by the different acting mechanisms of SAR (see Introduction)—inhibiting only Chk1 possibly results in the counter-regulatory effects of Chk2—or (2) by the (high) SAR and PRX concentration used—the proliferation-inhibiting effect of high-dose Chk-is (Figure 1b) might be causative for the mitigated radiation effects. The long-term proliferation-inhibiting effect of RT was also shown by staining Ki-76-positive cells at d28 (Figure 6). After combined RT and PRX therapy, a significant reduction in proliferating cells was shown by analyzing all RT-treated groups versus all sham-RT-treated groups (227 ± 49 to 191 ± 27 cells/FOV; *n* = 9 animals; *p* ≤ 0.01).

The potential toxicity of therapy was assessed based on mouse body weight (BW). The onset of bulky head was also monitored as a marker of growing tumors but was found to be too variable to reveal any differences between treatment groups. As tumor growth, depending on the direction of progression, did not always result in head bulging, this parameter was not suitable (Figure S1). After tumor inoculation, BW decreased by 5% at d1–2 due to anesthesia and intracranial surgery but stabilized at d3–4. Preliminary dose-finding experiments revealed that an intense treatment regime with PRX (5 × 10 mg/kg) and/or RT (5 × 1 Gy) leads to a strong decrease (10–20%) in BW, requiring analgesia, and it was, therefore, abandoned (Figure S1a). Lower doses of PRX (5 × 1 or 5 × 5 mg/kg), SAR, and RT (5 × 0.5 Gy) were well tolerated (Figure S1b).

Our data show, for the first time, the intracranial antitumor activity of SAR, although combinations of SAR with RT/chemotherapy have already been shown to delay tumor growth in head and neck and colorectal cancer mouse models [19,21]. For PRX, CNS penetration has already been proven [17,32] and is hereby supported in NSG mice. Several recently published clinical trials confirm PRX tolerability and efficiency [33,34,35,36,37,38,39,40,41], and one phase 1 trial examining the combination of PRX and chemotherapy in MB is currently active [18]. Also, in pediatric patients, PRX was under clinical examination in a phase 1 trial with 30 patients and revealed that PRX was well tolerated, with the only grade 3/4 regimen-related toxicity being hematologic, predominantly neutropenia [42]. Overlapping hematologic toxicities with genotoxic agents should be considered, making further dose-finding studies necessary. Data from MYCN-driven tumors implicate the combination of Chk-is not only with genotoxic therapies but also with DNA damage repair-affecting drugs, like PARP inhibitors [43], for improving treatment efficiency, warranting further investigations.

Limitations: Our study is limited by the number of animals and the drug/irradiation dose range used in the experiments. Similar to our in vitro results, there seems to be a very narrow optimal concentration range for Chk-is in combination with RT. Low concentrations show no activity, whereas higher concentrations interfere with RT, possibly because of their proliferation-inhibiting properties. The application of Chk-i at concentration levels that are non-toxic as a single agent and lower RT doses to allow for the detailed evaluation of (sub-) additive and synergistic effects are suggested but were not in the scope of this observational study. To evaluate if the demonstrated effects are specific to the SHH/*p53*-mut MB subtype, a second animal model with *p53*-wildtype tumors would be beneficial.

## 3. Materials and Methods

### 3.1. Cell Lines

UW228 (SHH/*p53*-mut MB) cells were kindly provided by Hendrik Witt (DKFZ, Heidelberg, Germany) and maintained in DMEM with 4.5 g/L glucose and 4 mM L-glutamine (Biozym, Hessisch Oldendorf, Germany, #880010). DAOY (SHH/*p53*-mut MB) cells were purchased from ATCC cell biology collection (Manassas, VA, USA) and maintained in MEM (Biozym #880124) and 2 mM L-glutamine (Sigma-Aldrich, Burlington, MA, USA, #G7513). Cell culture media were supplemented with 10% fetal calf serum (Sigma-Aldrich #F7524), 100 U/mL penicillin, and 100 U/mL streptomycin (Biozym #882082).

### 3.2. Drugs

For in vitro use, stock solutions of 10 mM prexasertib HCl (LY2606368; Selleckchem, Houston, TX, USA, #S7178) and 20 mM SAR-020106 (SYNkinase, Melbourne, Australia, #SYN-1189-M001) were prepared in DMSO (Sigma-Aldrich #D2650) and stored at −20 °C. Working solutions were diluted freshly in cell culture medium with a final DMSO concentration of 0.01% (PRX) or 0.005% (SAR).

### 3.3. Cell Culture Treatment and Assays

Cells were seeded and allowed to attach for 24 h. PRX (1–10 nM) or SAR (0.05–1 µM) was added 1 h prior to RT (single-dose). For fractionated experiments examining clonogenicity, half of a medium complemented with PRX (1–5 nM) or SAR (0.05–0.1 µM) was renewed daily. An X-ray machine (X-Strahl 200, Xstrahl GmbH, Ratingen) with dose rates of 1.3–1.9 Gy/min was used at 150 kV. Cell culture plates/flasks were placed on an 8 cm thick poly (methyl methacrylate) (PMMA) phantom; the focus-object distance (FOD) from the radiation source to the plate top was 30 cm. For the irradiation of single rows of cell culture plates, a house-made MCP96 alloy (1.8 cm) shielding devise (1 cm PMMA bottom) with variable irradiation gaps was applied. For all RT experiments, a 10 mA aluminum filter with a 6 mm half-layer value (150 kV) was used. Appropriate DMSO-treated and sham-irradiated controls were implemented.

Metabolic activity was detected using WST-1 reagent (Sigma-Aldrich #11644807001). Cell proliferation was measured using colorimetric BrdU cell proliferation ELISA (Sigma-Aldrich #11647229001). Cell death induced by apoptosis was detected by Annexin-V-FLUOS Staining Kit (Sigma-Aldrich #11988549001) and analyzed by fluorescence cytometry, as previously described [22]. The apoptotic cell fraction is defined as the percentage of Annexin-V-positive/propidium iodide-negative and Annexin-V-positive/propidium iodide-positive cells within all analyzed cells (100%). To determine the long-term survival of clonogenic cells, cells were seeded in 6-well plates and treated 24 h later on four consecutive days. At d14, adherent colonies were rinsed in phosphate-buffered saline, fixed in ice-cold ethanol/acetone (1:1) for 10 min, and stained with Giemsa solution for 5 min. Colonies ≥ 50 cells were counted manually under microscopic control. The surviving fraction (SF) was calculated by dividing the plating efficiency (PE) of treated cells by the PE of untreated cells, whereby PE is the number of colonies/seeded cells [44].

### 3.4. Fluorescence–Microscopic Analyses of DNA Damage in S Phase Cells

To determine DNA damage in the S/G2M cell cycle phase, double-staining of γH2AX protein and EdU incorporation were examined by immunofluorescence. Prior to cell fixation at 1, 24, or 72 h, cells were exposed to 10 µM EdU (Click-iT Plus EdU Flow Cytometry Assay Kit, Invitrogen #C10632) for 5 h. Staining of γH2AX protein was performed as previously described [22] with the following amendments: Prior to DAPI counter-staining, γH2AX-stained cells were fixed with EdU Click-iT Fixation solution for 15 min and washed once with PBS + 1% BSA + 0.5% Tween20 (wash buffer). Permeabilization with EdU Click-iT- Permeabilization and Wash solution for 15 min and one washing step with wash buffer followed. The EdU Click-iT Reaction cocktail was mixed, and cells were incubated according to the manufacturer’s instructions. Cells were washed three times, DAPI-counterstained, and mounted according to [22].

Microscopic images (BZ-9000; BZ-II Viewer; Keyence, Osaka, Japan) of at least 50 nuclei (DAPI) were taken for Alexa Fluor™ 488 (EdU) and Alexa Fluor™ 568 (γH2AX) using identical exposure parameters. Overlay pictures were analyzed using the hybrid cell count–fluorescence–double extraction application (BZ-II analyzer) and identical conditions.

### 3.5. Western Blot of DNA Damage Proteins

Western blot (WB) analysis of γH2AX, phospho-RPA, Histone H3, and GAPDH was adapted based on [22,45]. In brief, the cell suspension was washed twice with ice-cold PBS, resuspended in ice-cold assay buffer (RIPA) containing protease inhibitor (cOmplete^TM^, Roche, Basel, Switzerland), and sonicated 3 times (HTU SONI-130 MiniFIER, G. Heinemann, Schwäbisch Gmünd; 10 s on–20 s off; amplitude, 30%). For SDS-PAGE, 25 µg of isolated protein was loaded onto a 15% polyacrylamide gel together with a ScanLater^TM^ Protein Ladder (Molecular Devices). After electrophoresis, proteins were transferred to a PVDF membrane, blocked with TBST (Tris-buffered saline with polysorbate 20:20 mM Tris, 134 mM NaCl, 0.1% Tween 20; pH 7.6) + 2% BSA for 1 h, washed with TBST, and incubated with the following antibodies: mouse anti-phospho-Histone H2A.X (Ser139), clone JBW301, Millipore #05-636, 1:500; rabbit phospho-RPA32/RPA2 (Ser8) clone E5A2F, Cell Signaling Technology #54762, 1:1000; mouse Anti-GAPDH Loading Control Monoclonal Antibody (GA1R), Invitrogen Antibodies # MA5-15738, 1:1000; rabbit anti-histone H3 clone D1H2, XP^®^, Cell Signaling Technology #4499, 1:1000; secondary antibodies IRDye 680RD goat anti-mouse (#926-68070), 1:15000; and IRDye 800CW goat anti-rabbit (#926-32211), 1:8000 (Li-COR Biosciences).

### 3.6. Mouse Model, Treatment, and Imaging

Six- to eight-week-old female NSG™ mice (NOD.Cg-PrkdcscidIl2rgtm1Wjl/SzJ mice) were bred and housed as previously described [46]. All experiments were approved by the local authorities (Landesdirektion Sachsen, TVV36/19), including daily scoring for health criteria, e.g., body weight, bulky head, and neurological disorders. The orthotopic-patient-derived xenograft (PDX) SHH/*p53*-mutated, MYCN-amplified (BT084), and luciferase-transfected MB mouse model was created as described previously [46].

In this observational study (including preliminary dose-finding experiments), a total number of 34 mice were sacrificed. For the main experiments, three mice per treatment group and six mice per control group (placebo/sham-irradiated and irradiated only) were implemented. Randomization was performed by randomly selecting the mice to treatment groups. Human endpoints were defined as follows: loss of >20% body weight, strong neurological disorders, poor general condition and behavior, or intense bulked back of the head.

Mice were treated on five consecutive days (d7–11) after tumor cell transplantation (Figure 5a). Drug solutions were prepared daily using 40% DMSO in sterile water (PRX) or 10% DMSO + 5% Tween20 (Serva # 3747001) in 0.9% NaCl (SAR). Preliminary dose-finding studies were performed in this mouse model according to known pharmacokinetic mouse data and estimation from efficient in vitro concentrations: in other mouse models, a maximal SAR plasma concentration of about 50 µM (40 mg/kg; i.p.) and a maximal brain tumor concentration of 19–66 nM PRX (20 mg/kg; i.v.) were achieved [19,32]. We tested PRX concentrations of 10 mg/kg, 5 mg/kg, and 1 mg/kg s.c. Based on the known linear relationship of PRX bioavailability i.v.; 1 mg/kg might result in a brain concentration of 1–3 nM; 5 mg/kg in 5–15 nM and 10 mg/kg in 10–30 nM, which are in the scope of effective PRX concentrations in vitro. For SAR, the blood–brain penetration capability is still unknown; therefore, we used the highest published dose (40 mg/kg), which did not cause toxic adverse effects [19].

In the main experiments, 2 mL/kg of body weight were provided s.c. (PRX 1 or 5 mg/kg) or i.p. (SAR 40 mg/kg) 2 h prior to RT. Whole-brain irradiation (daily, 5×, d7–11) and bioluminescence imaging (BLI; weekly, d4 (prior treatment), d14, 21, 28, 35, 42) were performed using antagonizable narcosis, as described in [46]. For BLI, total flux (photons/second) was measured 13 min after luciferin injection with automatic exposure time.

### 3.7. Tissue Preparation and Staining

Mice were euthanized by CO2 anesthesia and decapitation 4 weeks after end of treatment. Tissue preparation, freezing, cryosectioning, and histological (H/E) or Ki-67 staining (tumor proliferative index; purified Mouse Anti-Ki-67, CloneB56, 250 µg/mL, BD Pharmingen #550609, 1:150) were performed as described previously [46]. The number of Ki-67-positive cell nuclei per field of view (FOV; 800 × 800 µm, ocular counting grid) was counted manually in three cryosections/mice under a Zeiss Axiolab microscope. As intratumoral proliferation was highly variable, evaluation was always conducted in the tumor area with the highest proliferation index.

### 3.8. Statistics

If not otherwise noted, statistical analysis between two treatment groups was conducted using a one-sided Student’s *t*-test using the Microsoft Excel 2016 software. *p*-values ≤ 0.05 (*; #) and ≤ 0.01 (**; ##) were considered statistically significant; *p*-values ≤ 0.001 (***; ###) were considered highly statistically significant. To validate normal distribution as a requirement for the parametrical *t*-test, we performed the Shapiro-Wilk test (IBM SPPS statistics version 29). Out of 141 tested treatment groups, only 3 failed (*p* ≤ 0.05). From this, we determined the normal distribution of all statistically analyzed data (WST-1 activity, BrdU incorporation, Annexin-V staining, Ki-67 staining).

## 4. Conclusions

In contrast to the very promising in vitro data showing strong antitumor activity regarding the metabolism, proliferation, clonogenic survival, and DNA damage of combined Chk-i/RT versus RT alone, the in vivo experiments showed differential results. Although we demonstrated, for the first time, the intracranial antitumor activity of the Chk1-specific inhibitor SAR in mice, the benefit of the integration of Chk-is in SHH/*p53*-mut MB therapy could not be proven. The Chk-i concentration seems to be especially critical, as high-dose Chk is partly compromise the RT effect on tumor growth, possibly by inhibiting proliferation. The implications of this study might also hold true for other MB subgroups, especially the MYC-driven Group 3 MB, warranting further investigations.

## Data Availability

The original contributions presented in this study are included in the article/Supplementary Material. Further inquiries can be directed to the corresponding author.

## Supplementary Materials

The following supporting information can be downloaded at https://www.mdpi.com/article/10.3390/ijms26062577/s1.

## Abbreviations

The following abbreviations are used in this manuscript:

ATR	Ataxia telangiectasia and Rad3-related protein
BLI	Bioluminescence imaging
BW	Body weight
Chk	Checkpoint kinase
Chk-i	Checkpoint kinase inhibitor
HNSCC	Head and neck squamous cell carcinoma
MB	Medulloblastoma
NSG mouse	Nod-SCID gamma mouse
OS	Overall survival
PDX	Patient-derived xenograft
PE	Plating efficacy
PRX	Prexasertib
RT	Irradiation; radiation therapy
SAR	SAR-020106
SF	Surviving fraction
SHH/*p53*-mut	SHH-activated and *TP53*-mutated
WB	Western blot
