# Supplementary material for: Antitumor Activity of Radiation Therapy Combined with Checkpoint Kinase Inhibition in SHH/p53-Mutated Human Medulloblastoma
Source: Int J Mol Sci. 2025 Mar 13;26(6):2577. doi: 10.3390/ijms26062577 (PMC11942233; doi:10.3390/ijms26062577)
Supplement: Supplementary file 1 [file ijms-26-02577-s001.zip › ijms-3445115-supplementary.pdf]

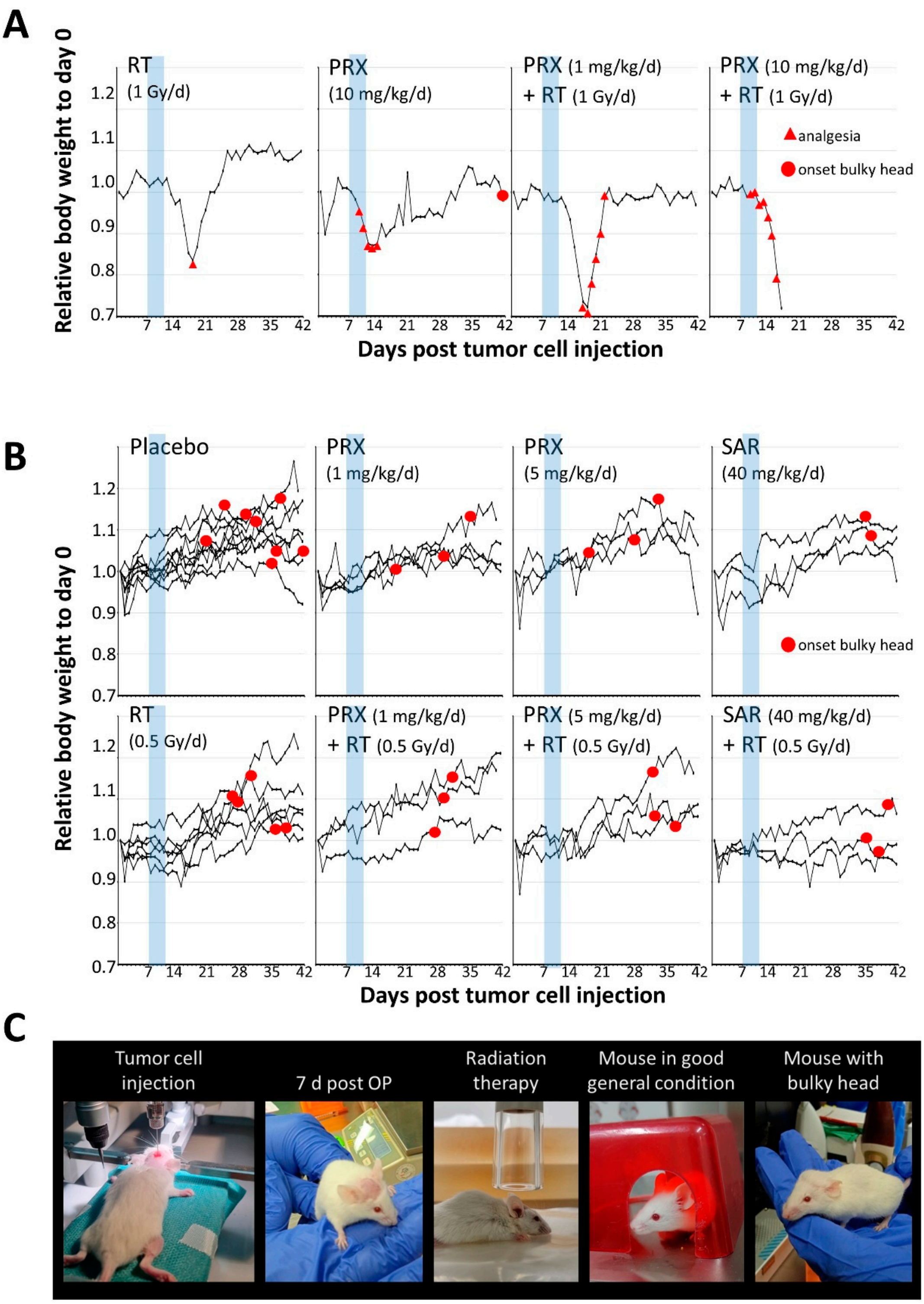

Supplementary Figure 1: Evaluation of potential toxic treatment effects by body weight of SHH/p53-mut MB-bearing mice before/during/after fractionated therapy (5x, daily) with PRX or SAR and RT. Data were assessed during the daily health monitoring procedure of mice used for in vivo tumor growth experiments (figure 4). Daily BW of single mouse (each line represents one mouse) over time normalized to d0. Onset of bulky head and necessity of analgesia is indicated by red dots or triangle (according to scoring criteria authorized by the Landesdirektion Sachsen (TVV36/19)). Treatment window is shaded in blue. (A) High-dose treatments, which had do be abandoned for toxicity. Dose

finding experiments showed massive reduction of BW at low dose PRX (1 mg) if combined with RT (1 Gy), and at high-dose PRX (10 mg) treatments even requesting euthanasia. (B) Conditions used for the main experiments. IR with 0.5 Gy combined with reduced PRX concentration (max. 5 mg/kg/d) showed no toxicity; neither did SAR (40 mg/kg/d).
